# Editorial for the Special Issue on Security and Sensing Devices for Healthcare Technologies

**DOI:** 10.3390/mi12091028

**Published:** 2021-08-27

**Authors:** Syed Aziz Shah, Naeem Ramzan, Muhammad Ali Imran, Qammer Hussain Abbasi

**Affiliations:** 1 Research Centre for Intelligent Healthcare, Coventry University, Coventry CV1 5RW, UK; 2 School of Computing, Engineering & Physical Sciences, University of the West of Scotland, Paisley PA1 2BE, UK; Naeem.Ramzan@uws.ac.uk; 3 James Watt School of Engineering, University of Glasgow, Glasgow G12 8QQ, UK; Muhammad.Imran@glasgow.ac.uk; 4 Communication Sensing and Imaging Group, James Watt School of Engineering, University of Glasgow, Glasgow G12 8QQ, UK; Qammer.Abbasi@glasgow.ac.uk

Micro-/nano-scaled structures, materials, and devices enable the continuous monitoring of human physical activities and behaviors, as well as physiological and biochemical parameters during daily life. The most commonly measured data include vital signs such as heart rate, blood pressure, and body temperature, as well as blood oxygen saturation, posture, and physical activities through the use of an electrocardiogram (ECG), ballistocardiogram (BCG), and other devices. Potentially, wearable photo or video devices could provide additional clinical information. Wearable devices can be attached to shoes, eyeglasses, earrings, clothing, gloves, and watches and may evolve to be skin-attachable devices. Sensors can be embedded into the environment, such as chairs, car seats, and mattresses. Healthcare is undergoing a rapid transformation from a traditional hospital- and specialist-focused approach to a distributed patient-centric approach. Advances in several technologies fuel this rapid transformation of healthcare vertically. Among the various technologies, communication technologies have enabled us to deliver personalized and remote healthcare services.

The Special Issue consists of five contributions in the area of security and sensing devices for healthcare technologies.

In the paper [1], A Fiber Ring Laser Sensor with a Side Polished Evanescent Enhanced Fiber for Highly Sensitive Temperature Measurement, the application of secured sensing devices is presented that uses the ring laser sensor leveraging fabricated sensing system to record the temperature of the physical environment, exploiting a refined evanescent enhanced fiber structure equipped with an isopropanol, having a high sensitivity. This sensing system uses a wavelength division multiplexer with an interconnected pump of the smallest size with a high gain medium. The polarization controller is put in the temperature sensing unit to modulate the state of the polarity. This system works on the light when strikes via the single fiber tuned with a basic mode and reaches the sideways polished units, presenting mismatch among the core diameters of the fiber sensor. The results obtained from the experimental performed have a smaller wavelength (in nm), having a threshold of around 400 mW. It is noticed that the transmitting wavelength of the signal is highly dependent on the lowest spectral loss. The system also demonstrated that the visibility of the interference signal pattern is hugely identified using the high intensity of the cavity mode of the proposed system [1].

In the paper [2], Design and Evaluation of a Flexible Dual-Band Meander Line Monopole Antenna for On- and Off-Body Healthcare Applications, a flexible dual-bank meander lien monopole antenna used for on- and off-body person monitoring is introduced. It is another application, specifically for the healthcare sector, using micro-/nano-scaled structures. The proposed antenna is a low-complexity flexible miniature and is evaluated using different subjects, in different conditions such as when the subject body was (i) dry, (ii) wet, (iii) hot, and (iv) cold with two different operating frequency bands with different body sizes of the subjects for on- and off-body and of body operations. The novel antenna introduced used a planar monopole-loaded asymmetrical inverted slots and a ground plane truncated at the bottom made up of the copper material. The antenna was exited using a microstrip connected to the monopole surface. The antenna designed primarily works in dual band (S-band and C-band) and is examined at an optimum distance of 10 mm from the skin layer.

In the paper [3], Full Ground Ultra-Wideband Wearable Textile Antenna for Breast Cancer and Wireless Body Area Network Applications, an ultra-wideband antenna used for body area network was presented, focusing on acquiring breast images. The full ground UBW antenna covers multiple challenges that the previous antenna presented. For instance, it works in near-field and far-field as opposed to the existing one that only works in close proximity to the high dielectric medium of the human body. The proposed antenna for breast cancer imaging is a flexible miniaturized antenna using a substrate that has a high dielectric constant. The proposed antenna uses traditional patch that improved the gain and bandwidth, exploiting a technique known as photonic band gap structures in conjunction with substrate integrated waveguide. This work also investigated its performance in free space and radiation characteristics to identify several tumors present in the breast.

In the paper [4], High Gain Triple-Band Metamaterial-Based Antipodal Vivaldi Multiple Input Multiple Output (MIMO) Antenna for 5G Communications, the authors present a dual-band MIMO antenna with a high isolation. This antenna was designed to address the existing challenges in the field of MIMO antennas. In this research work, the foundation design had one antipodal spiral antenna integrated with seven elements made up of metamaterial and were investigated specifically for 5G applications. This antenna was also interlinked and connected with a micro-electro-mechanical system, implying that this antenna can be reconfigured very easily and can be easily deployed in the 5G communication network.

In the paper [5], Portable Device for Quick Detection of Viable Bacteria in Water, an easy-to-use, lightweight device that can be used to detect water quality level at rapid pace is presented. The device exploits different chemicals such as dimethylthiazol, diphenyltetrazolium, and phenazine methosulfate when they are used in bacterial cell membranes. The device is formed of four key parts, namely a light source, cubetter hold, sensor, and a lightweight microcontroller. A light-emitting diode of white color lights up when a water sample is tested in the specific holder. The light is then absorbed by the water sample using reflection, refraction, and scattering phenomena using a six-channel device, which is essentially capable of obtaining signal at different wavelengths. The average cost of the proposed water testing device is around $15. The primary reason of being low-cost and lightweight is due to the fact that the sensors and LED drivers make it cost-effective and easily deployable.

## References

[1] Lin W., Liu Y., Shao L., Vai M.I. (2021). A Fiber Ring Laser Sensor with a Side Polished Evanescent Enhanced Fiber for Highly Sensitive Temperature Measurement. Micromachines.

[2] Ali S.M., Sovuthy C., Noghanian S., Ali Z., Abbasi Q.H., Imran M.A., Saeidi T., Socheatra S. (2021). Design and Evaluation of a Flexible Dual-Band Meander Line Monopole Antenna for On- and Off-Body Healthcare Applications. Micromachines.

[3] Mahmood S.N., Ishak A.J., Saeidi T., Soh A.C., Jalal A., Imran M.A., Abbasi Q.H. (2021). Full Ground Ultra-Wideband Wearable Textile Antenna for Breast Cancer and Wireless Body Area Network Applications. Micromachines.

[4] Saeidi T., Ismail I., Noghanian S., Alhawari A.R.H., Abbasi Q.H., Imran M.A., Zeain M.Y., Ali S.M. (2021). High Gain Triple-Band Metamaterial-Based Antipodal Vivaldi MIMO Antenna for 5G Communications. Micromachines.

[5] Liao Y.-H., Muthuramalingam K., Tung K.-H., Chuan H.-H., Liang K.-Y., Hsu C.-P., Cheng C.-M. (2020). Portable Device for Quick Detection of Viable Bacteria in Water. Micromachines.

